# A Malignant Masquerade: Angiosarcoma Presenting as an Impending Rupture of an Abdominal Aortic Aneurysm

**DOI:** 10.7759/cureus.81233

**Published:** 2025-03-26

**Authors:** Koki Tamaoka, Takeshi Shimamoto, Kazuhisa Sakamoto, Makoto Takehara

**Affiliations:** 1 Cardiovascular Surgery, Hamamatsu Rosai Hospital, Hamamatsu, JPN

**Keywords:** abdominal aortic aneursym (aaa), diagnostic delay, primary angiosarcoma, vascular neoplasms, vascular surgeon

## Abstract

Primary tumors arising from within the arterial wall are extremely uncommon and often present at advanced stages, making timely intervention challenging. Surgeons need to be aware of these rare conditions, as early detection and treatment can significantly improve patient outcomes. Angiosarcoma of the abdominal aorta is a particularly rare and aggressive malignancy, often resembling an aneurysm at risk of rupture. A 59-year-old woman presented with sudden onset of severe abdominal pain and was diagnosed with a 42 mm saccular abdominal aortic aneurysm, which was urgently repaired under the presumption of impending rupture. Intraoperatively, we found the aneurysm to be notably more fragile than a typical abdominal aortic aneurysm, while the rest of the aorta appeared healthy. This discrepancy raised suspicion of an atypical lesion, prompting us to resect the aneurysm and submit it for pathology. Subsequent immunohistochemical staining confirmed angiosarcoma, enabling timely referral for oncological management. Angiosarcoma is often difficult to diagnose based on computed tomography findings alone, so any atypical appearance of the aorta should raise suspicion of this malignancy, even in emergent surgery. This case shows the important role of careful intraoperative assessment and pathological examination in guiding definitive management.

## Introduction

Angiosarcoma is a rare and aggressive malignant tumor arising from endothelial cells, most commonly affecting the skin, soft tissues, or visceral organs [1,2]. Primary angiosarcomas originating within major arteries, particularly the abdominal aorta, are extremely uncommon, with only a limited number of cases reported in the literature [3].

Due to its non-specific symptoms and frequent appearance of conventional aneurysms, diagnosis can be challenging, often leading to delayed or inadequate treatment. Moreover, angiosarcoma is notorious for its aggressive behavior and early metastatic potential, emphasizing the need for high clinical suspicion and prompt intervention [4].

Treatment strategies typically involve surgical resection, which may be combined with chemotherapy and radiotherapy, depending on the extent of the disease [2]. Complete surgical excision offers the best chance of local control, but long-term outcomes remain uncertain due to the high likelihood of recurrence or metastasis [4].

In this report, we present the case of an abdominal aortic angiosarcoma initially misinterpreted as an impending rupture of the abdominal aorta aneurysm (AAA), underscoring the importance of recognizing atypical intraoperative findings and performing immediate histopathological evaluation to guide timely oncological management.

## Case presentation

A 59-year-old woman with no significant past medical history arrived at the emergency department, complaining of acute abdominal and back pain that had intensified over the preceding several hours. She described the pain as sharp and progressive, with occasional radiation to her flank. On initial examination, her vital signs showed mild hypotension (blood pressure 100/65 mmHg) and tachycardia (heart rate 100 beats per minute). Laboratory test was largely unremarkable apart from a slightly elevated white blood cell count (Table 1).

**Table 1:** Preoperative blood test results PT: prothrombin time; INR: international normalized ratio; APTT: activated partial thromboplastin time

Test	Results	Normal range
White blood cells (× 10^3^/µL)	9.4	3.3-8.6
Hemoglobin (g/dL)	14	11.6-14.8
Hematocrit (%)	42.6	35.1-44.4
Platelet (× 10^4^/μL)	26.9	15.8-34.8
PT-INR	1.11	0.9-1.1
APTT (seconds)	32.8	24-34
Fibrinogen (mg/dL)	350	200-400
C-reactive protein (mg/dL)	0.06	0-0.14
Creatine phosphokinase (U/L)	51	41-421
Sodium (mmol/L)	138	138-145
Potassium (mmol/L)	4	3.6-4.8
Chloride (mmol/L)	101	101-108
Urea nitrogen (mg/dL)	15	8. 0-20. 0
Creatinine (mg/dL)	0.36	0. 46-0.79
Aspartate aminotransferase (U/L)	21	13-30
Alanine aminotransferase (U/L)	16	10-42
Lactate dehydrogenase (U/L)	176	124-222
Total bilirubin (mg/dL)	1.2	0.4-1.5
Total protein (g/dL)	7.3	6.6-8.1
Albumin (g/dL)	4.5	4.1-5.1

**Figure 1:**
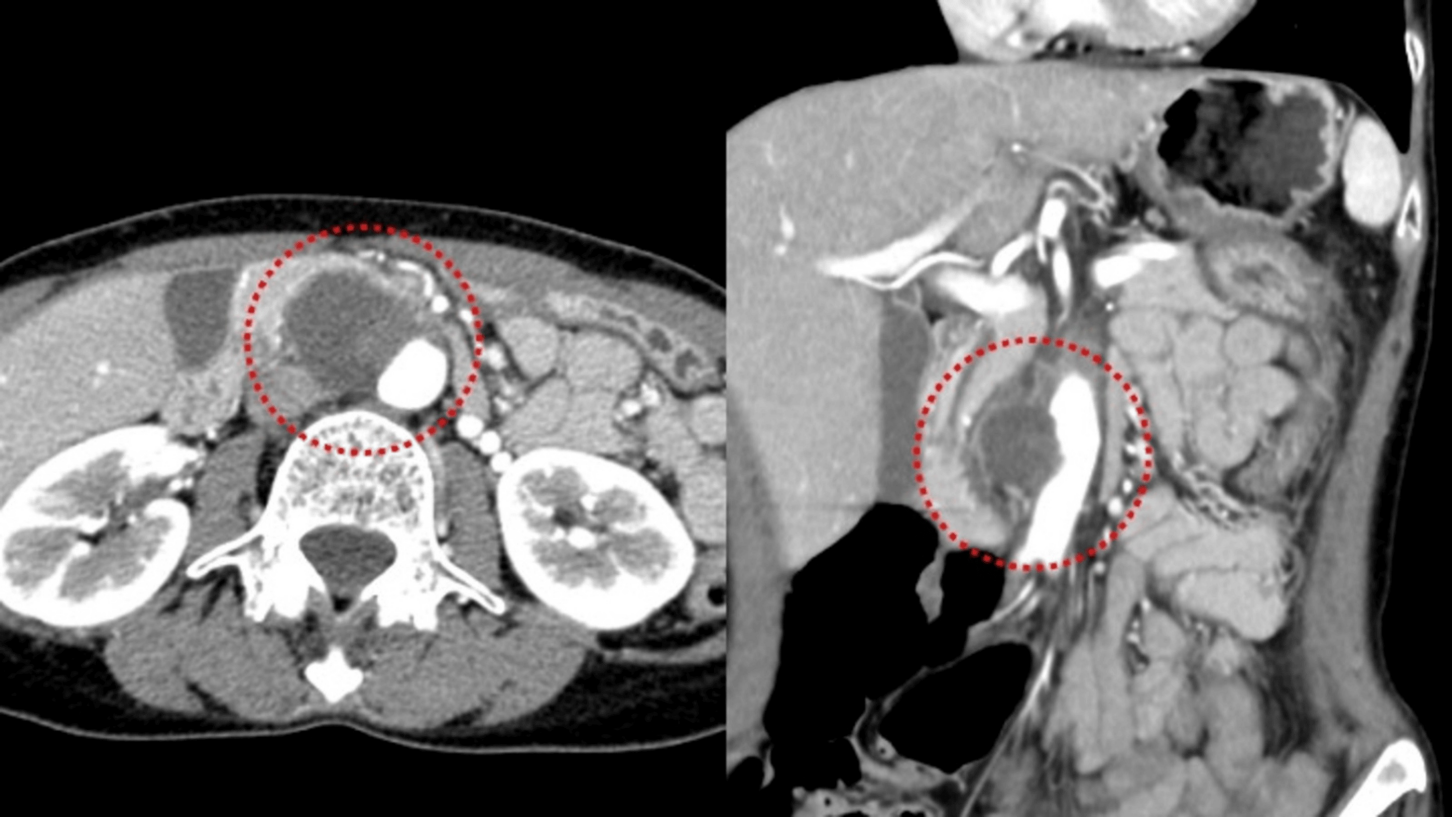
Preoperative contrast-enhanced CT The red dashed line encloses the abdominal aortic aneurysm, which was initially diagnosed as an impending rupture based on clinical presentation and imaging findings.

Contrast-enhanced computed tomography (CT) revealed a 42-mm saccular aneurysm arising from the abdominal aorta and extending toward the duodenum (Figure 1). Importantly, the unaffected portions of the aorta showed no calcification or atheromatous changes typically seen in degenerative aneurysms, suggesting that this lesion was not simply a conventional AAA.

**Figure 2:**
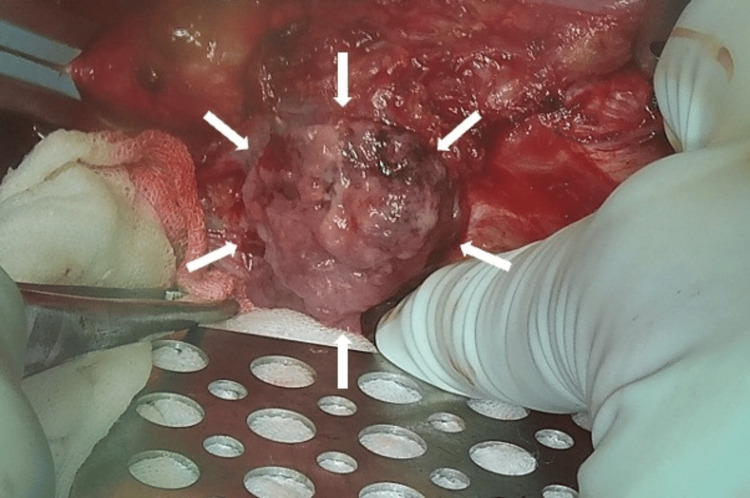
Intraoperative view of the mass The aneurysmal segment appeared an unusually fragile wall and a tumor-like appearance, rather than the typical degenerative changes seen in standard aortic aneurysms. At this stage, a pseudoaneurysm was considered due to its atypical characteristics.

Given the rapid escalation of her pain and concerns over an impending rupture, emergent surgical intervention was performed. Under general anesthesia, a midline abdominal incision was made. On entering the peritoneal cavity, no hemoperitoneum or gross hemorrhage was detected. We encountered an aneurysmal mass arising from the aorta and adherent to the duodenum (Figure 2).

Compared with a typical AAA, the mass wall structure appeared more fragile, and we initially suspected it might be a pseudoaneurysm. Nonetheless, the rest of the aortic wall appeared relatively healthy, with no signs of prior focal dissection. We suggested that this lesion represented an atypical process rather than a standard degenerative aneurysm.

Consequently, we performed an extensive resection of the aneurysmal segment and replaced it with a 14-mm tubular Dacron graft. The procedure proceeded without major complications, and the patient’s hemodynamics remained stable throughout.

**Figure 3:**
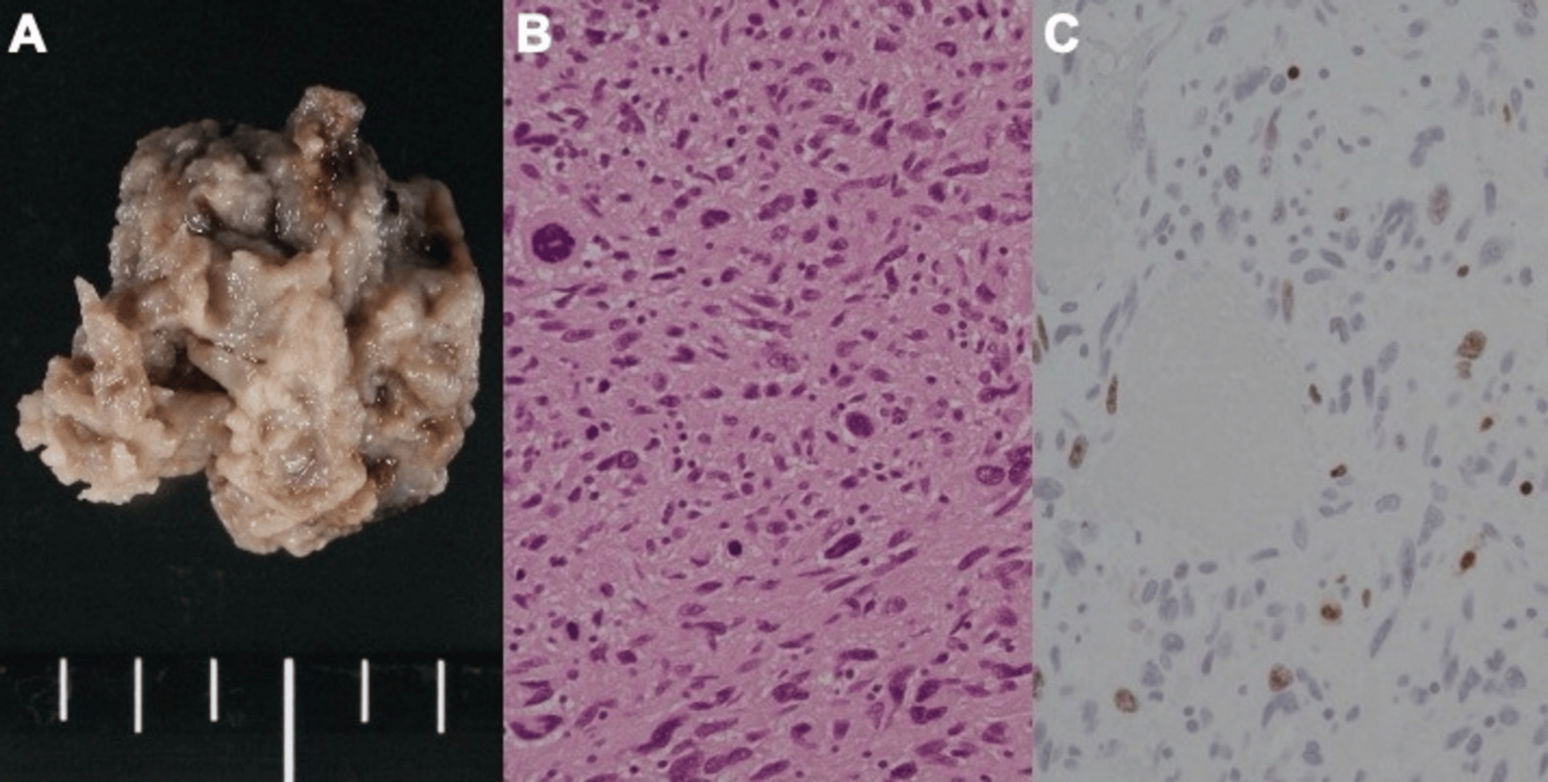
Macroscopic view of the resected aneurysmal specimen(A) and histopathological examination reveals scattered enlarged, irregular nuclei (B). Ki-67 immunostaining shows a labeling index of approximately 15%, which is relatively low for a angiosarcoma; however, pronounced nuclear atypia and areas of necrosis indicate a malignant tumor.

Postoperatively, her course was uneventful, and she exhibited good recovery with no significant issues. Histopathological examination of the resected specimen, supported by immunohistochemical staining, demonstrated a high Ki-67 labeling index and nuclear atypia consistent with angiosarcoma (Figure 3).

**Figure 4:**
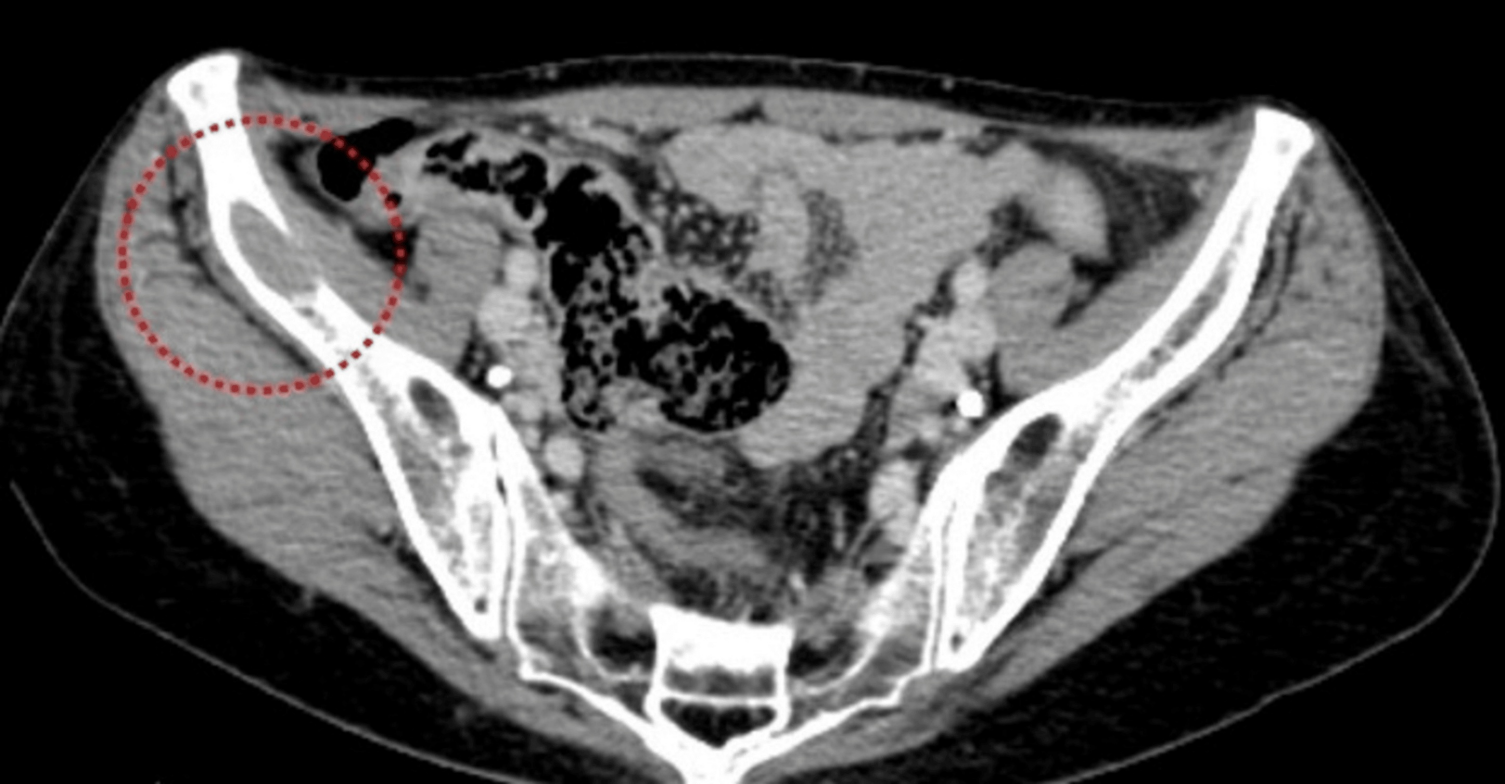
Retrospective review of the preoperative CT A low-density lesion (red circle) is visible in the iliac bone, suggesting possible metastatic involvement associated with the primary angiosarcoma.

A subsequent review of her preoperative CT images revealed a subtle low-density region near the iliac bone, raising the possibility of metastatic disease (Figure 4). She was referred for oncologic evaluation, and further treatment, including chemotherapy, was initiated after a multidisciplinary discussion.

## Discussion

This case presented with acute abdominal and back pain, and her preoperative imaging suggested an impending rupture of AAA [5]. Such a clinical scenario typically prompts urgent surgical intervention based on the assumption that a degenerative or atherosclerotic aneurysm may soon rupture. However, upon direct visualization of the aneurysm with laparotomy, the aneurysmal segment appeared atypical. Therefore, the specimen was sent for pathological analysis. This led to the unexpected diagnosis of angiosarcoma, underlining the importance of considering rare malignant etiologies when encountering aneurysms with unusual features.

Had endovascular aneurysm repair been performed, the opportunity for immediate histopathological sampling would have been missed, potentially delaying the detection of angiosarcoma [6]. Radiologically, angiosarcomas can mimic conventional aneurysms, and their malignant features often remain subtle on contrast-enhanced CT. Consequently, early recognition of the tumor based solely on imaging is challenging [7].

However, if the lesion is different from the typical characteristics of an atherosclerotic aneurysm, such as a non-calcified, open repair allows direct evaluation and definitive diagnosis based on histology, facilitating timely initiation of oncologic treatment.

Equally important is the need for a thorough preoperative evaluation of potential metastatic disease. In this patient, a retrospective review of the CT indicated a low-density area near the iliac bone, suggesting possible metastasis. Identifying distant involvement beforehand can guide both the surgical approach and subsequent oncologic therapies [8,9].

Angiosarcoma is known for its aggressive behaviour and rapid progression [1], so comprehensive imaging is important in planning the best possible management strategy.

Overall, this case demonstrates the need to maintain a broad differential diagnosis when faced with an atypical aneurysm, to select a surgical approach that allows for immediate pathological evaluation when warranted, and to thoroughly evaluate for metastatic spread. These strategies can improve outcomes in patients with this rare yet aggressive malignancy [10,11].

## Conclusions

Primary angiosarcoma of the abdominal aorta is rare and can be mistaken for AAA impending rupture. Even in emergency situations, suspicious intraoperative or radiological findings require immediate pathological evaluation. Early recognition and timely initiation of oncological treatment may improve outcomes in this aggressive malignancy.
